# Cost-Benefit Analysis of Vaccination Strategies to Prevent Mother-to-Child Transmission of the Hepatitis B Virus Using a Markov Model Decision Tree

**DOI:** 10.3389/fpubh.2022.662442

**Published:** 2022-06-21

**Authors:** Nan Yang, Lei Lei, Yiyu Meng, Naitong Zhou, Lizheng Shi, Ming Hu

**Affiliations:** ^1^West China School of Pharmacy, Sichuan University, Chengdu, China; ^2^School of Public Health and Tropical Medicine, Tulane University, New Orleans, LA, United States

**Keywords:** hepatitis B virus, mother-to-child transmission (MTCT), vaccination, hepatitis B immunoglobulin (HBIG), cost-benefit analysis, Markov model, decision tree

## Abstract

**Objectives:**

Currently, in China, several strategies exist to prevent mother-to-child transmission (MTCT) of the hepatitis B virus (HBV). These include providing Hepatitis B vaccination and hepatitis B immunoglobulin (HBIG) injection with different types of administration and dosages. The aim of this study is threefold: first, to evaluate the economic viability of current hepatitis B vaccination strategies for preventing MTCT from a public health policy perspective; second, to optimize the current immunization strategy for preventing perinatal transmission of the HBV; and third, to offer policy options to the National Health Commission in China.

**Methods:**

To simulate the disease outcome for the entire life of newborns infected with HBV, a Markov model with eight possible health states was built by using TreeAge Pro 2011 software. In the present study, the model parameters were probability and cost, which were extracted from literature and calculated using Microsoft Excel 2013. The optimal immunization strategies were identified through cost-benefit analyses. A benefit-cost ratio (BCR) > 1 indicated that the strategy had positive benefits and *vice versa*. A one-way sensitivity analysis was used to investigate the stability of the results.

**Results:**

From a public health care system perspective, we evaluated the economic viability of 11 strategies in China. For all 11 strategies, the BCR was > 1, which indicated that the benefits of all the strategies were greater than the costs. We recommended strategy number 9 as being optimal. In strategy number 9, babies born to hepatitis B surface antigen (HBsAg)-positive mothers were given an HBIG (200 IU) within 24 h of birth and three injections of hepatitis -B vaccine (20-μg each) at 0, 1, and 6 months, and the strategy had a BCR of 4.61. The one-way sensitivity analysis revealed that the full vaccination coverage and effective rates of protection were two factors that greatly influenced the BCR of the different prevention strategies; other factors had little effect.

**Conclusion:**

The benefits of all strategies were greater than the costs. For decision-making and application, the strategy should be based on local socio-economic conditions so that an appropriate immunization strategy can be selected.

## Introduction

Hepatitis B virus (HBV) infection is a public health problem worldwide. It causes acute and chronic liver disease and puts people at a high risk of death from cirrhosis or hepatocellular carcinoma (1). Chinese patients account for approximately one-third of the 350 million individuals infected with HBV worldwide, which also causes a substantial economic burden (2).

As the major route of HBV transmission, mother-to-child transmission (MTCT) accounts for 40–50% of chronic infections (3). Effective intervention measures are urgently needed to prevent MTCT of HBV. In China, since 2002, every newborn is required to be vaccinated against HBV at birth, 1 month, and 6 months. The prevalence of women of childbearing age in China (4) being infected with HBV is documented to be 7.18%.

Hepatitis B immunoglobulin (HBIG) is a substance collected from healthy human blood. It is administered widely to provide passive prophylactic immunity against HBV infection (5–7). The use of an immunization strategy based on a combination of the hepatitis B vaccine and HBIG to block MTCT has achieved significant clinical effects: low prevalence of intrauterine infection, higher protection, and fewer adverse events (5–8). In China, several strategies for preventing MTCT of HBV involve hepatitis B vaccination and HBIG with different administration routes and doses (8). In Europe and the US, a few cost-benefit studies have been conducted to assess their strategies for preventing MTCT of HBV through combined immunization using HBIG (9). However, those results cannot be directly applied to China because the prevalence of HBV, economic conditions, the susceptibility of people, and modes of transmission vary in different countries. Although economic evaluations of strategies for preventing MTCT of HBV were undertaken in China, a long-term assessment is still lacking (10). Therefore, it is necessary to evaluate the economic viability of the current hepatitis B vaccination strategies for preventing MTCT in China from a public health policy perspective. The approach can optimize the current immunization strategy for preventing MTCT of HBV and offer suggestions to the National Health Commission in China.

## Materials and Methods

### Target Population

Eleven strategies for preventing MTCT of HBV in China were analyzed. The target population of the 11 strategies was newborns of pregnant women who were positive for the surface antigen of the hepatitis B virus (HBsAg) and double-positive for HBsAg/ hepatitis B e antigen (HBeAg).

There were four types of prevention strategies. The first strategy involved pregnant women receiving an injection of HBIG in the late pregnancy period and newborns being vaccinated with the hepatitis B vaccine. The second strategy comprised newborns being vaccinated with HBIG and the hepatitis B vaccine simultaneously. The third strategy involved newborns being vaccinated only with the hepatitis B vaccine. The fourth strategy comprised pregnant women receiving an injection of HBIG late into their pregnancy and newborns being vaccinated with HBIG and the hepatitis B vaccine simultaneously. The dose of the hepatitis B vaccine could be 5, 10, or 20 μg, and the babies were inoculated three times (0, 1, and 6 months) (Table 1).

**Table 1:** Strategies for preventing MTCT of HBV.

**Strategy number**	**HBsAg-positive pregnant women/mother**			**Newborns**			
	**time of dose (IU HBIG) of injection**			**time and dose (μg HepB) of injection**			
	**28 weeks** **of gestation**	**32 weeks** **of gestation**	**36 weeks** **of gestation**	**Within 24 h** **of birth**	**0 month** **after birth**	**1 month** **after birth**	**6 months** **after birth**
1	200	200	200	-	5	5	5
2	200	200	200	-	10	10	10
3	200	200	200	100	5	5	5
4	-	-	-	100	10	10	10
5	-	-	-	200	5	5	5
6	-	-	-	200	10	10	10
7	-	-	-	-	5	5	5
8	-	-	-	-	10	10	10
9	-	-	-	200	20	20	20
10	100	100	100	100	5	5	5
11	200	200	200	100	10	10	10

After the implementation of a certain strategy, vaccinated subjects were divided into “vaccinated” and “not vaccinated” groups. People in the vaccinated group were further divided into “immune-protected” and “not immune-protected” groups, in which each subject with hepatitis B surface antibody (anti-HB) titer >10IU/mL was considered to be immune-protected. Newborns who were not vaccinated or not immune-protected were considered to be susceptible and likely to be infected with HBV.

### Evaluation Method

A cost-benefit analysis is a basic analytical method that compares the costs and benefits in monetary terms (11). In the present study, “costs” referred to the cost of immunization under the different prevention strategies and the economic loss due to failed inoculation of the hepatitis B vaccine in the susceptible population. The “benefits” were costs saved due to effective prevention of HBV infection and related diseases. According to the cost and benefit of different immunization strategies, we calculated the benefit-cost ratio (BCR) of each immunization strategy. A BCR >1 indicated that the strategy had positive benefits and *vice versa*. The larger the BCR, the greater the benefits of the strategy. The formula for calculating the BCR is:


$$\begin{array}{l} \frac{B}{C}=\frac{\sum_{t=0}^{n}B_{t}{\left(1+r\right)}^{-t}}{\sum_{t=0}^{n}C_{t}{\left(1+r\right)}^{-t}}, \end{array}$$

where B_t_ is the benefit of t years, C_t_ is the cost of t years, t is the research time, and r is the discount rate.

### Modeling

#### Modeling Method

**Figure 1:**
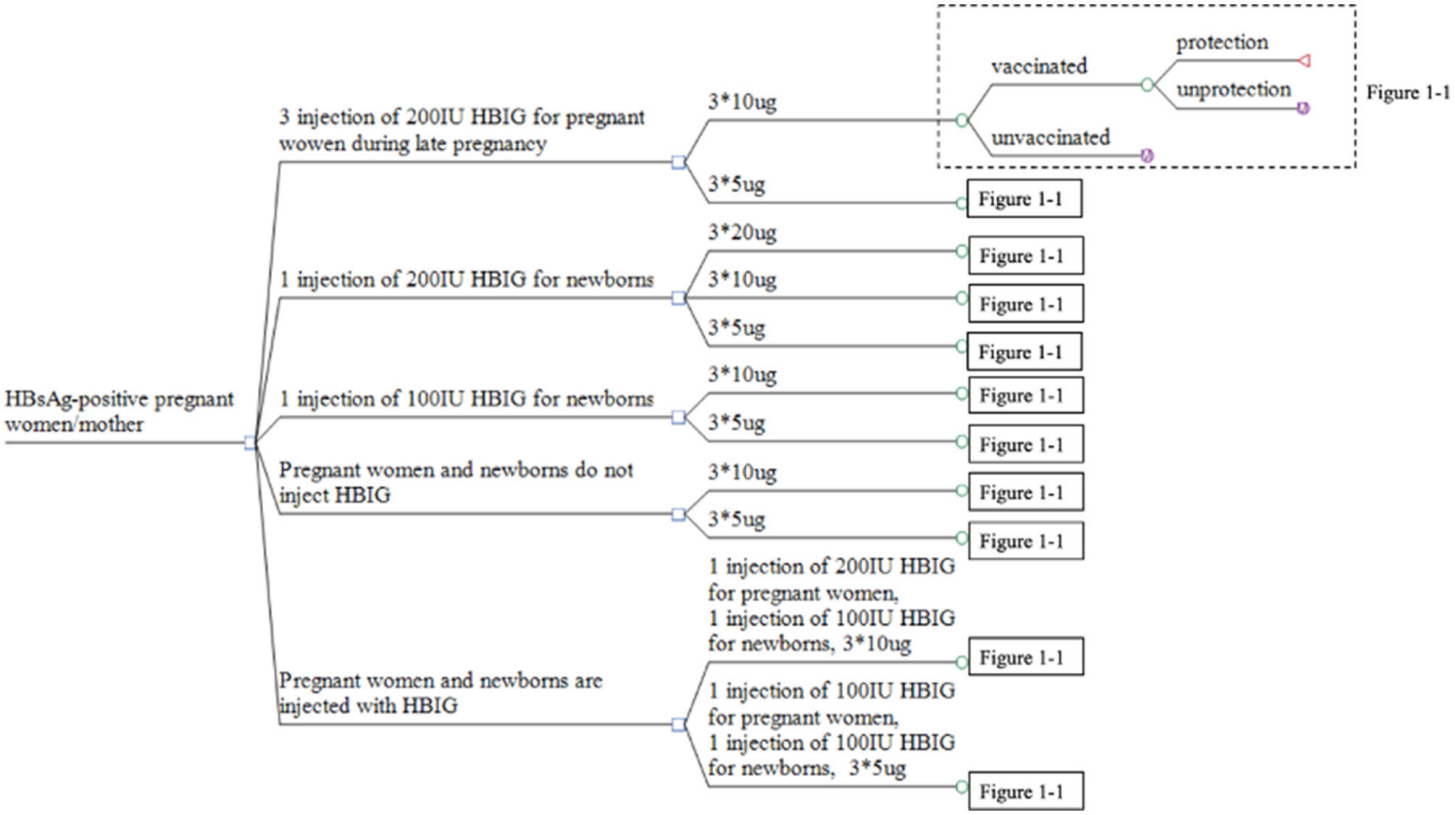
Decision tree for the strategies used to prevent MTCT of HBV. *Represents time of inoculation.

Based on the 11 strategies in China to prevent MTCT of HBV, we constructed a multi-level decision tree model (Figure 1). To simulate the long-term outcome of the disease on the entire life of newborns infected with HBV, a Markov model was built using TreeAge Pro 2011. This Markov model decision tree was created to undertake a cost-benefit analysis of the 11 strategies.

#### Markov Model Decision Tree Structure

**Figure 2:**
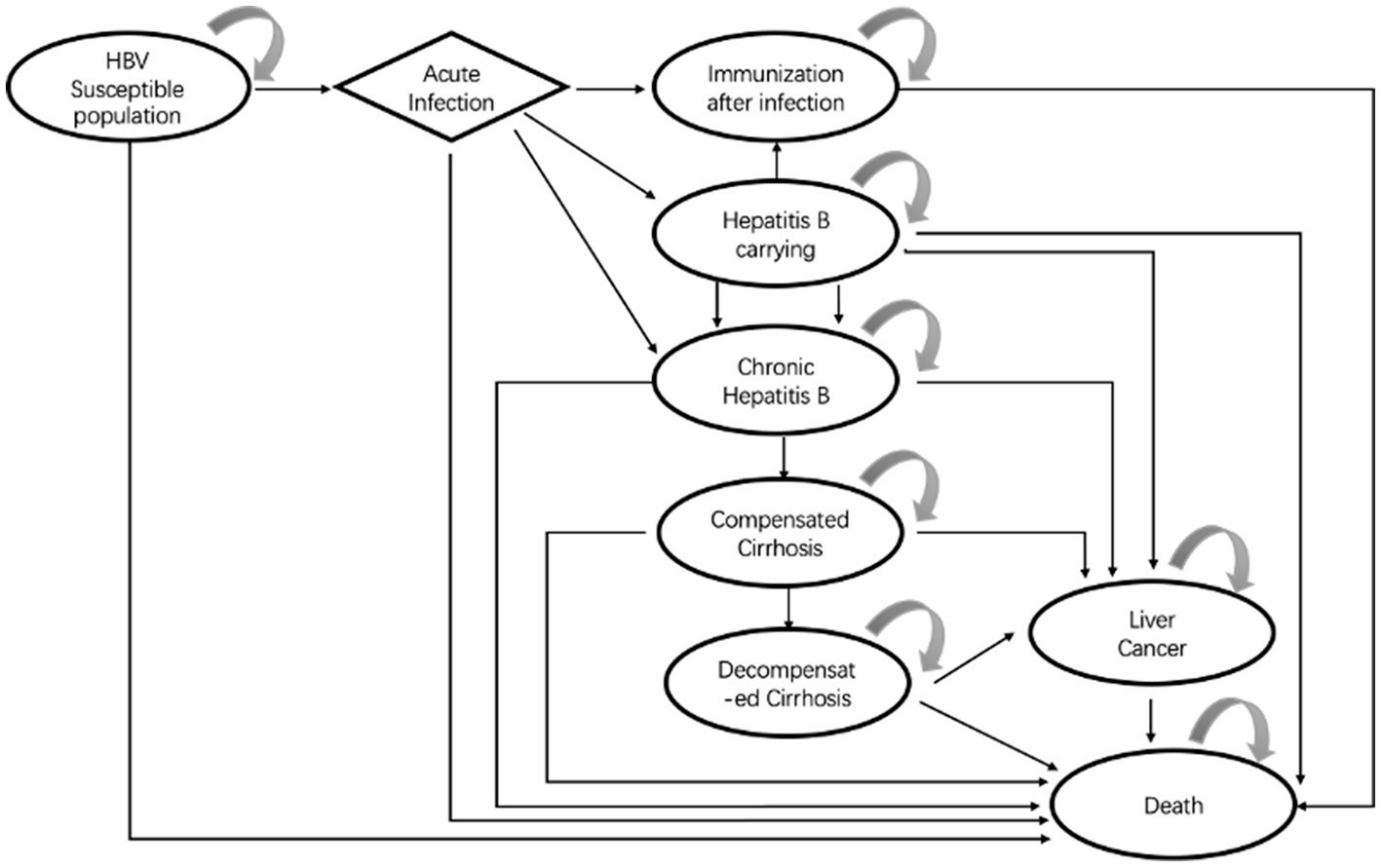
Markov diagram after HBV infection.

The Markov model decision tree in the study was constructed based on the disease course of hepatitis B and the effect of an intervention on disease progression. We refereed vaccination guidelines, reviews, and the literature for the prevention and treatment of HBV infection published up to 31 January 2018. Finally, with available data, we constructed a Markov model with eight possible health states (Figure 2) HBV susceptible population; acute infection; immunization after infection; HBV carriage; chronic hepatitis B disease (HBD); compensated cirrhosis; decompensated cirrhosis; liver cancer; and death. After that, we also consulted clinical and pharmacoeconomic experts, and the rationality of the model structure and assumptions were approved by the experts.

#### General Assumptions About the Model

Based on available data and the actual situation, four main hypotheses were made during the construction and analysis of the Markov model decision tree of strategies for preventing MTCT of HBV: The first hypothesis was that the immunity obtained by a hepatitis B-susceptible population by vaccination or HBV infection would be lifelong. The second hypothesis was that we considered only the prevention strategy which affected the vaccination target directly [shown by the positive conversion rate (PCR) of hepatitis B surface antibody (anti-HBs)] but not the immune barrier formed by the immunity of the population. The third hypothesis was that the cost of adverse reactions of HBV infection and HBIG administration was not considered with regard to their high safety, low probability of adverse reactions, and low risk of death (12). The fourth hypothesis was that in addition to the respective rates of death from severe hepatitis B disease (HBD), decompensated cirrhosis, or liver cancer, death from natural causes was used for all other health states in the Markov model.

### Parameters

The parameters of the model in this study were probability and cost. The probability parameters were as follows: effective rates of protection (ERP)/PCR of anti-HBs; full vaccination coverage (FCV), the annual rate of HBV infection among the susceptible population; and probability of disease transition after HBV infection. The cost parameters were as follows: the vaccination cost for each strategy for preventing MTCT of HBV; the economic burden of HBD and related diseases; age-specific life expectancy; and discount rate.

When estimating parameters, we first selected results from national surveys or authoritative health institutions [e.g., the Chinese Center for Disease Control and Prevention (CDC)]. If such results were unavailable, meta-analysis results of relevant studies or large-sample randomized controlled trials were used as references. If the methods stated above were unworkable, expert interviews or references to relevant literature from China were used.

#### ERP/PCR of Anti-HBs

We systematically reviewed the clinical literature published in English or Chinese using the database of SinoMed, China National Knowledge Infrastructure, Chongqing VIP Information, and Pubmed from 31 January 2018. To estimate the ERP, we used the following keywords: “hepatitis B vaccin^*^ OR HBV vaccin^*^ OR HBIG OR hepatitis B immune globulin, and mother-to-fetus OR mother-to-child OR MTCT OR vertical transmission”. The target population was limited to people from China.

Through literature review, 15 articles were included. We extracted the ERP/PCR of anti-HBs for each strategy from literature or estimated value (Table 2).

**Table 2:** ERP/PCR of anti-HBs and FVC for 11 immunization strategies.

**Strategy number**	**Effective rate of** **protection/anti-Hbs** **positive conversion rate (%)**	**Value range** **(%)**	**References**	**Full vaccination coverage** **(%)**
1	88.1	87.3–88.9	(13–15)	82.5
2	88.86	77.5–96.87	(16–18)	80.5
3	83.23	80–95.3	(19–21)	91
4	83.44	71.1–90.83	(18, 22–24)	90
5	83.15	82–90	-	88
6	90.3	85–95	(25)	87
7	69.29	56.3–77.4	(13, 15, 19, 20, 26)	95
8	72.04	63.4–80.28	(18, 22, 24, 26, 27)	95
9	96.8	77–97	(21, 25)	86
10	91.78	92–93	(21)	76
11	87.46	82–92	(28)	74

*ERP, Effective rate of protection; PCR, positive conversion rate; FVC, full vaccine coverage. Since it was not directly available from the literature, the ERP for strategy number 5 was estimated based on the actual situation and other strategies*.

#### FVC

We estimated the FVC for each strategy by referencing it to the results from the report of the national planned immunization review in China (2004), the epidemiological survey on national hepatitis B in China (2006), and literature reports from China on preventing MTCT of the HBV in China. These sources provided the relevant information from the provinces of Guangdong, Jiangxi, Shanghai, and Hubei (25, 29–34). We took into account other influencing factors (35) (e.g., difficulty during inoculation and acceptance of different strategies) and determined the FVC based on expert opinion (Table 2).

#### Annual Infection Rate of the HBV Among Susceptible Population

We obtained data on HBV infection in the Chinese population before hepatitis B vaccination from the National Hepatitis Serum Epidemiology Survey in China (1992). Then, we simulated the infection rate of HBV in China through a revised simple catalytic model using SAS (36). The hyperbolic function was:

f(a) = 0.0158 + 0.4056/(a + 1),

where f(a) represents the annual infection rate of a population and a represents age.

Our study was conducted based on the data from HBsAg-positive pregnant women and their newborns. Liaw et al. (37) reported that 10–20% of newborns delivered from HBsAg positive pregnant women who did not have the hepatitis B vaccine *postpartum* became carriers of chronic HBV infection (37). Edmunds et al. (38) reported that 90% of newborns became carriers of chronic HBV infection after perinatal infection (38). Hence, a newborn delivered from an HBsAg-positive pregnant woman has a 10–20% probability of carrying chronic HBV infection, with an infection rate during the perinatal period (from birth to 1 month of age) ranging between 11.11 and 22.22%. In this study, we assumed that the infection rate of a newborn delivered from an HBsAg-positive pregnant woman from 0 year to 1 year would be the same as that of the general population. According to the formula used for the model, the infection rate of the general population from 0 year to 1 year may be between 22.93 to 38.45%. We estimated that the infection rate of an HBsAg-positive woman could be 34.04–60.67% from 0 year to 1 year; the standard value in the model was 50%. The annual infection rate for other ages was estimated by the formula used to calculate the new infection rate.

#### Probability of Disease Transition After HBV Infection

The transition probability of each Markov state was obtained from the publicly available research. If multiple studies had the same probability of disease transition, then we took the mean value. The maximum value and minimum value of each transition probability reported in the literature were taken as the value range of sensitivity analysis.

The probability of disease transition after HBV infection in the Markov model was based on the relevant literature in China (Table 3).

**Table 3:** Status and probability of disease transition after HBV infection.

**Health status**	**Disease progression**	**Age** **(years)**	**Probability**	**Range**	**References**
**Acute Infection**					
	Symptomatic				
		0–1	0.01	±50%	(39)
		1–5	0.1	±50%	
		>5	0.3	±50%	
	Severe Hepatitis B	<20	0.01	±50%	(39)
		>20	0.005	±50%	
	Hepatitis B Carrier	<1	0.89	±50%	(20)
		>1			
	Death due to severe Hepatitis B	<15	0.63	±50%	(39)
		15–45	0.8	±50%	
		>45	0.93	±50%	
	Chronic hepatitis B		0.04	±50%	(40–42)
		Perinatal period	0.9	±50%	
		0–1	0.3	±50%	
		1–5	0.25	±50%	
		6–19	0.06	±50%	
		≥20	0.04	±50%	
				
**Hepatitis B carrier**					
	Natural immunity after infection	<30	0.008	±50%	(43)
		30–39	0.011	±50%	
		40–49	0.017	±50%	
		>50	0.018	±50%	
	Chronic Hepatitis B					
		<30	0.009	±50%	(43)
		30–39	0.014	±50%	
		40–49	0.028	±50%	
		>50	0.02	±50%	
	Liver Cancer				(44–47)
		0–19	0.0005	±50%	
		20–30	0.002	±50%	
		≥40	0.0061	±50%	
**Chronic hepatitis B**					
	Hepatitis B Carrier		0.03	0–0.07	(43, 45, 46, 48, 49)
	Compensated Cirrhosis				(33, 44–47, 50)
		0–19	0.0081	±50%	
		20–30	0.02	±50%	
		≥40	0.02	±50%	
	Liver Cancer				(33, 44–47, 50)
		0–19	0.003	±50%	
		20–30	0.005	±50%	
		≥40	0.0064	±50%	
**Compensated Cirrhosis**					
	Decompensated Cirrhosis		0.052	0.026–0.1	(39, 43, 50–56)
	Liver Cancer		0.029	0.0028–0.1	(37, 39, 43, 48–60)
**Decompensated cirrhosis**					
	Liver Cancer		0.05	0.0082–0.091	(14, 20, 37, 48–51, 57, 61–69)
	Death		0.1889	0.1–0.39	(39, 48–50, 52–55, 57–60, 62, 65, 66)
**Liver cancer**					
	Death		0.4971	0.3–0.8025	(39, 48, 49, 52–56, 59, 60, 67)

#### Cost Input

This study was based on a public healthcare system perspective. The cost of preventing MTCT of HBV comprised direct costs and indirect costs. Direct costs included the expense of HBIG or HBV vaccination, transportation and storage (including cold chain) expenditure, service and material expenditure during inoculation, and transportation costs for immunization of pregnant women. It was calculated based on the costs of medical services at Chinese non-profit medical institutions indicated in the research literature. Indirect costs included the compensation for absence from work for the pregnant women and their chaperones.

In China, the whole process of immunization against HBV involves three stages: (a) the provincial CDC is responsible for the procurement, storage, and transportation of the hepatitis B vaccine within the province; (b) the municipal, district, and national CDCs are responsible for the distribution, transportation, and storage of the vaccine within each jurisdiction; and (c) immunization clinics at all levels are responsible for vaccination. According to research articles assessing inoculation expenditures (vaccine procurement, cold-chain management, administrative expenses, and labor payment) of five hospitals, 92 CDCs, 177 community healthcare centers, and 476 village clinics in Shanghai, Shandong, Hunan, and Gansu Provinces, the cost of one injection was US$ 0.35–0.50 (14, 68). The price of the hepatitis B vaccine and HBIG were determined according to the purchase price paid by provincial CDCs.

Transport expenses for pregnant women injected with HBIG (which included bus, taxi, and personal cars) and indirect costs (which included compensation for absence from work for pregnant women and their companions) were extracted from literature (61) (Table 4).

**Table 4:** Cost for 11 immunization strategies (in USD).

**Item**		**Baseline** **(USD)**	**Range** **(USD)**
Cost for different items of inoculation	Hepatitis B vaccine: 5ug	0.71	0.57–0.85
	Hepatitis B vaccine: 10ug	1.00	0.85–1.14
	Hepatitis B vaccine: 20ug	3.55	3.41–3.70
	HBIG: 100IU	21.33	19.91–22.75
	HBIG: 200IU	42.65	41.23–44.08
	Cold-chain	0.35	0.34–0.35
	Direct non–medical cost	1.84	1.42–2.13
	Indirect cost	0.57	0.43–0.71
Cost for 11 immunization strategies	Immunization Strategy 1	144.27	136.61–164.83
	Immunization strategy 2	145.12	137.47–151.90
	Immunization strategy 3	29.69	26.66–32.39
	Immunization strategy 4	30.54	27.51–33.24
	Immunization strategy 5	51.01	47.97–53.71
	Immunization strategy 6	51.87	48.83–54.56
	Immunization strategy 7	8.01	6.42–9.30
	Immunization strategy 8	8.85	7.28–10.16
	Immunization strategy 9	59.51	56.50–62.23
	Immunization strategy 10	101.91	95.90–110.19
	Immunization strategy 11	166.70	157.70–174.99

#### Disease Burden Related HBV Infection

The economic burden of HBD and related diseases included the direct economic burden and indirect economic burden, which were based on a systematic review of the literature. The “direct economic burden” referred to consumed social and economic resources with regard to the treatment of HBD and related diseases, which included direct medical costs and nonmedical costs. The “indirect economic burden” referred to the indirect economic losses caused by HBD and related diseases to the society.

We identified 12 studies focusing on HBD burden and extracted the information listed in Table 5 (63, 69–78). The studies involved 7,409 respondents from Zhejiang, Guangdong, Shanghai, Shanxi, Gansu, Jiangsu, and Shandong Provinces and reported the burden of HBD and related diseases.

**Table 5:** Burden of hepatitis B disease and related diseases (in USD).

**Health Status of** **Hepatitis B disease**	**Direct economic** **burden**	**Indirect economic** **burden**	**Annual** **economic burden**	**Range**
Chronic hepatitis B	2,898.20	660.87	3,559.06	1,614.49–5,141.29
Compensated cirrhosis	4,154.83	906.25	5,061.07	2,259.81–8,108.36
Decompensated cirrhosis	5,456.73	1,914.66	7,391.39	4,136.03–14,359.19
Liver cancer	5,724.14	4,676.39	10,400.55	1,880.60–33,988.50
Acute infection	2,943.73	1,136.10	4,079.83	2,025.82–8,177.58
Severe hepatitis B	9,164.76	2,465.85	11,630.61	4,001.41–14,988.95

#### Life Expectancy, Age-Specific Mortality, and Discount Rate

Age-specific life expectancy was a parameter required for the simulation of the Markov model after the HBV-susceptible population had become infected and it determined the number of cycles of the Markov model. It was determined using the Global Health Observatory data available on the World Health Organization's (WHO) website (79). Based on the *Chinese Statistical Yearbook* (2016), we determined the life expectancy of the Chinese population to be 76 years (80). The number of cycles in the Markov model was set at 76. Mortality caused by severe HBD ensued from acute infection, decompensated cirrhosis, and liver cancer. We determined the mortality associated with other health statuses in the Markov model based on age-specific mortality in the general population under natural conditions. The natural mortality rate of the whole population from 0 to 74 years adopted the age-specific mortality rate of Chinese residents of small- and medium-sized cities according to the *Health Statistics Yearbook of China* (2011) (81). Linear interpolation was used to obtain the mortality per year.

The effect of immunization had long-term benefits, and the outcome of the disease after HBV infection was also a long-term process. To eliminate the time effect on the currency, the costs and benefits in our study were discounted. The discount rate of 5% was calculated based on similar studies conducted in China. The range of values of the discount rate used in the sensitivity analysis was 3–6%, as recommended by the World Bank and similar studies conducted in the US, Sweden, and the United Kingdom.

### Sensitivity Analyses

#### One-Way Sensitivity Analyses

We evaluated the influence of each parameter in the Markov model decision tree that we constructed in the study and investigated the stability of the results. We adopted an item-by-item substitution method. In this process, one parameter was changed in the model and the other parameters were kept constant to analyze the main parameters in the model within the range of each value. The main parameters in the study were: PRC of anti-HBs and the FVC for each immunization strategy; immunization costs; expenses of each health state in the Markov model after HBV infection; and discount rate.

#### Threshold Analyses

The threshold analyses were also undertaken with a one-way sensitivity analysis. The objective was to calculate the value of the parameters if the optimal strategy changed.

## Results

### Decision-Making Results of Vaccination Strategies to Prevent MTCT of HBV in China

We input the related parameters into the constructed decision tree using TreeAge Pro 2011. The cost, benefit, net benefit, and BCR under different immunization strategies were obtained (Table 6).

**Table 6:** Cost-benefit analysis for vaccination strategies to prevent MTCT of the hepatitis B in China (in USD).

**Strategy number**	**Cost/person**			**Benefit/person**	**Net benefit/person**	**BCR**
	**Cost of** **vaccination**	**Cost of HBV** **Infection Due to** **Immune Failure**	**Total** **cost**			
1	144.18	1,258.09	1,402.27	3,347.35	1,945.08	2.39
2	145.04	1,311.06	1,456.10	3,294.38	1,838.28	2.26
3	29.67	1,117.31	1,146.98	3,488.14	2,341.16	3.04
4	30.52	1,146.94	1,177.46	3,458.51	2,281.05	2.94
5	50.98	1,235.55	1,286.53	3,369.90	2,083.37	2.62
6	51.84	987.37	1,039.21	3,618.09	2,579.58	3.48
7	8.01	1,573.88	1,581.89	3,031.56	1,449.66	1.92
8	8.87	1,453.57	1,462.44	3,151.87	1,689.44	2.16
9	59.51	771.51	831.02	3,833.95	3,004.35	4.61
10	101.90	1,393.02	1,494.92	3,212.43	1,717.50	2.15
11	166.74	1,624.78	1,791.52	2,980.66	1,189.19	1.66

Table 6 shows that strategy number 9 was optimal with a BCR of 4.61. Strategy 3 and strategy 6 were less optimal options. All 11 vaccination strategies to prevent MTCT of HBV in China had a BCR >1; so all the prevention strategies were economically feasible. However, the most suitable vaccination strategy might be different for different regions based on local health needs and economic conditions.

### Sensitivity Analyses

Taking the optimal strategy 9 as an example, for each respective parameter, minimum value, maximum value, baseline value, and the class-mid value were given as input into the Markov model for sensitivity analyses. Parameter assignments for sensitivity analyses of the Markov model decision tree are shown in Table 7.

**Table 7:** Parameter assignment for sensitivity analysis of the decision tree combined with the Markov model (in USD).

**Parameter**	**Minimum value**	**Class-mid value 1**	**Baseline value**	**Class-mid value 2**	**Maximum value**
Full vaccination coverage	74%	80%	86%	90.5%	95%
BCR	2.42	3.25	4.61	6.40	9.85
Effective rate of protection	69.29%	83.05%	96.8%	-	-
BCR	1.42	2.39	4.61	-	-
Cost of vaccination (USD)	56.50	58.00	59.51	60.87	62.23
BCR	4.63	4.62	4.61	4.61	4.60
Discount rate	3%	4%	5%	5.5%	6%
BCR	4.76	4.69	4.61	4.57	4.52
**Cost for health status in the Markov model (in USD)**					
Acute hepatitis B	2,025.82	3,052.82	4,080.29	6,129.40	8,178.51
Chronic hepatitis B	1,614.49	2,586.78	3,559.47	4,350.67	5,141.87
Compensated cirrhosis	2,259.81	3,660.86	5,061.64	6,585.46	8,109.28
Decompensated cirrhosis	4,136.02	5,764.36	7,392.23	10,876.52	14,360.82
Liver cancer	1,880.60	6,141.27	1,0401.73	22,197.05	33,990.37
Severe hepatitis B	4,001.43	7,816.90	1,1631.93	13,311.29	14,990.65
BCR	4.23	4.49	4.61	4.69	4.75

**Figure 3:**
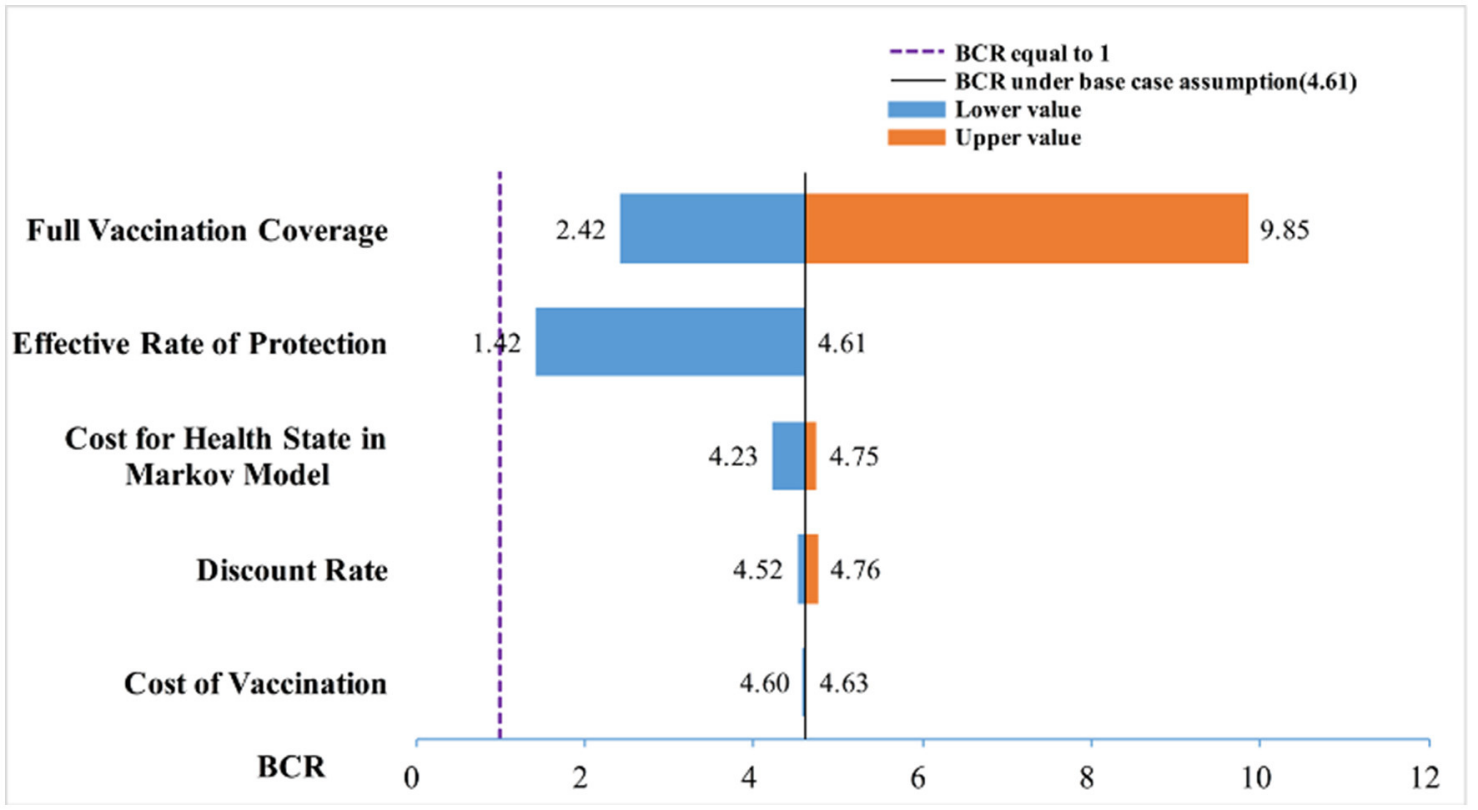
Tornado diagram for the one-way sensitivity analysis for the BCR.

The one-way sensitivity analysis showed that some model parameters had a substantial impact on the BCR of the study. As seen in Figure 3, the longer the bar, the more sensitive the parameter is to the constructed model. A slight change in the parameter caused a significant change in the BCR and *vice versa*. The most sensitive factor in the Markov model was the FVC of each immunization strategy, followed by the FVC and the annual cost of HBD and related diseases after HBV infection. Other factors had little effect on our model. For the practical application of an immunization strategy preventing MTCT of HBV, close attention should be paid to factors with high sensitivity. The efficiency of the resource input and BCR could be increased with the best use of factors with high sensitivity.

## Discussion

The prevention of MTCT of HBV has been widely studied by researchers all over the world. With various preventive strategies currently available, choosing an effective and economic method to prevent MTCT is a common challenge faced by health institutions and scholars. We used TreeAge Pro 2011 to create a decision tree combined with Markov model, determined its parameters to simulate the disease process after HBV infection, and calculated the cost and benefit of different strategies to prevent MTCT of HBV. From a public healthcare system perspective, we recommended strategy number 9 as being optimal in China. Comparing the current immunization programs and policies in the country (82), we offered our suggestions to the national health administration in China. Our one-way sensitivity analysis revealed that the FVC and ERP of different prevention strategies had a great influence on the BCR, whereas other factors had little effect. These results are consistent with the results of other similar studies (83).

## Limitations

There are several limitations to be acknowledged. First, we assumed that the immunization obtained by a hepatitis B-susceptible population by vaccination or HBV infection would be lifelong. A proportion of vaccinated newborns had a poor response or gradual erosion of vaccine with time (84). The issue of revaccination and re-infection of HBV has not been considered in this study. Second, comprehensive literature reviews and scientific methods were used to estimate the values of the FVC and ERP, but further studies are needed. Third, HBIG and the hepatitis B vaccine were assumed to be safe and reliable without side effects. The cost of adverse reactions of HBV infection and HBIG administration was not considered. However, as a blood product, HBIG has certain safety issues, which must be considered during inoculation. This may have an impact on the accuracy of the results.

## Conclusion

From a public healthcare system perspective, we evaluated the economic viability of 11 immunization strategies in China. For all 11 strategies, the BCR > 1 meant that the benefits of all strategies were greater than the costs. We recommended strategy number 9 as being optimal in China. In strategy number 9, babies born to HBsAg-positive mothers are given an HBIG (200 IU) within 24 h of birth and three injections of the hepatitis B vaccine (20-**μ**g each) at 0, 1, and 6 months, and the BRC is 4.61 for this case. For decision-making and application, the strategy should be based on the social and economic conditions of different regions so that an appropriate immunization strategy can be selected.

## Data Availability Statement

The original contributions presented in the study are included in the article/supplementary material, further inquiries can be directed to the corresponding author.

## Author Contributions

NY designed the study, interpreted data, and drafted the manuscript. LL and YM collected and analyzed data and drafted the manuscript. NZ collected and interpreted the data. MH designed the study, interpreted the data, and revised the manuscript critically for important intellectual content. LS revised the manuscript critically for important intellectual content. All authors read and approved the final manuscript before submission.

## Funding

This research was supported by the Key R&D Projects of the Science and Technique Foundation in Sichuan Province (2021YFS0145) and a grant from the 111 Project (Overseas Expertise Introduction Project for Discipline Innovation, B18035).

## Conflict of Interest

The authors declare that the research was conducted in the absence of any commercial or financial relationships that could be construed as a potential conflict of interest.

## Publisher's Note

All claims expressed in this article are solely those of the authors and do not necessarily represent those of their affiliated organizations, or those of the publisher, the editors and the reviewers. Any product that may be evaluated in this article, or claim that may be made by its manufacturer, is not guaranteed or endorsed by the publisher.

## Abbreviations

HBV: hepatitis B virus
MTCT: mother-to-child transmission
HBIG: hepatitis B immunoglobulin
HBsAg: surface antigen of the hepatitis B virus
HBeAg: hepatitis B e antigen
anti-HB: hepatitis B surface antibody
PCR: positive conversion rate
HBD: hepatitis B disease
ERP: effective rates of protection
FCV: full vaccination coverage
BCR: benefit-cost ratio
CDC: Chinese Center for Disease Control and Prevention
USD: United States Dollar.
